# Differential Diagnosis of Skin Ulcers in a *Mycobacterium ulcerans* Endemic Area: Data from a Prospective Study in Cameroon

**DOI:** 10.1371/journal.pntd.0004385

**Published:** 2016-04-13

**Authors:** Laurence Toutous Trellu, Patrick Nkemenang, Eric Comte, Geneviève Ehounou, Paul Atangana, Didier Junior Mboua, Barbara Rusch, Earnest Njih Tabah, Jean-François Etard, Yolanda K. Mueller

**Affiliations:** 1 Geneva University Hospitals, Geneva, Switzerland; 2 Médecins Sans Frontières, Geneva, Switzerland; 3 Centre Pasteur Cameroon, Yaounde, Cameroon; 4 Yaounde Central Hospital, Yaounde, Cameroon; 5 National Yaws, Leishmaniasis, Leprosy and Buruli ulcer Control Program against, Ministry of Public Health, Yaounde, Cameroon; 6 Epicentre, Paris, France; 7 Institut de Recherche pour le Développement (IRD) UMI 233 –INSERM U 1175 – Montpellier University, Montpellier, France; University of Tennessee, UNITED STATES

## Abstract

**Background:**

Clinical diagnosis of Buruli ulcer (BU) due to *Mycobacterium ulcerans* can be challenging. We aimed to specify the differential diagnosis of skin lesions in a BU endemic area.

**Method:**

We conducted a prospective diagnostic study in Akonolinga, Cameroon. Patients presenting with a skin ulcer suspect of BU were included. *M*. *ulcerans* was detected using swabs for Ziehl-Neelsen staining, PCR and culture. Skin punch biopsies were taken and reviewed by two histopathologists. Photographs of the lesions were taken and independently reviewed by two dermatologists. Final diagnosis was based on consensus, combining the results of laboratory tests and expert opinion.

**Results/ Discussion:**

Between October 2011 and December 2013, 327 patients with ulcerative lesions were included. Median age was 37 years (0 to 87), 65% were males, and 19% HIV-positive. BU was considered the final diagnosis for 27% of the lesions, 85% of which had at least one positive laboratory test. Differential diagnoses were vascular lesions (22%), bacterial infections (21%), post-traumatic (8%), fistulated osteomyelitis (6%), neoplasia (5%), inflammatory lesions (3%), hemopathies and other systemic diseases (2%) and others (2%). The proportion of BU was similar between HIV-positive and HIV-negative patients (27.0% vs. 26.5%; p = 0.940). Half of children below 15 years of age were diagnosed with BU, compared to 26.8% and 13.9% among individuals 15 to 44 years of age and above, respectively (chi2 p<0.001). Children had more superficial bacterial infections (24.3%) and osteomyelitis (11.4%).

**Conclusion:**

We described differential diagnosis of skin lesions in a BU endemic area, stratifying results by age and HIV-status.

## Author Summary

In some areas of Africa, Australia or Japan, a specific skin infection presents as a wound which progressively increases in size in children and people of any age. The agent which causes this infection is named *Mycobacterium ulcerans*, close to the tuberculosis agent. This wound, also named Buruli ulcer (BU), may be confused with other common cutaneous diseases. During two years in Akonolinga, Cameroon, we evaluated the wounds of all patients who presented with suspected BU. This wound presentation was most frequently recorded in young children and males. Buruli ulcer was indeed the most frequent diagnosis in this area. However, with the help of laboratory and radiological techniques, we found that many of those patients not diagnosed with BU were suffering from: vascular insufficiency (older persons), benign superficial infections and bone infections (children). This observation is important and should help improve the diagnosis and treatment of patients with skin ulcers in Africa.

## Introduction

Infection by *M*. *ulcerans*, more commonly known as Buruli Ulcer (BU), is a neglected tropical disease that has been reported in33 countries in Africa, the Americas, Asia and the Western Pacific [1]. West Africa has the highest number of cases, found mainly in Benin, Cameroon, Côte d’Ivoire, Democratic Republic of the Congo, and Ghana. *M*. *ulcerans* infection generally begins with a localised, itchy skin lesion which evolves into localized (nodule) or diffuse (oedema) swelling, or as an indurated plaque. Over time, the lesions progressively develop into craters that result in potentially massive ulcers with undermined edges. Without proper treatment, the scarring process can lead to contracture deformities and movement limitations. [2, 3]

The laboratory technique most commonly used in the field for BU diagnosis is direct examination by microscopy using a Ziehl-Neelsen (ZN) stained smear in search of Alcohol- and Acid-Fast bacilli, with a sensitivity of ZN of approximately 40% [4, 5]. The same staining technique can also be used for non-ulcerated plaques using fine needle aspiration [6]. Culture of *M*. *ulcerans* is a procedure with a sensitivity rate of 20–60% [5]. Polymerase Chain Reaction for insertion sequence IS2404 is very specific and currently the most sensitive diagnostic method with a sensitivity of 85% [7]. Histopathology is rarely used in the field, as the method requires adapted tools for sample collection as well as specific knowledge to both prepare and interpret the slides.

Cases of painless ulcerated plaques with undermined edges, classically described as BU [1], are believed to be relatively easy to clinically diagnose in endemic regions; the difficulty lies in diagnosing lesions in their early stages or when located in parts of the body where vascular ulcers are also common. In a retrospective study carried out in Ghana, the specificity of clinical diagnosis was estimated to be 94% [8]. Nevertheless, without laboratory testing to confirm the findings, diagnostic errors are probably highly under-estimated.

Various forms of BU can be mistaken for other types of ulcers found in tropical areas. Among the most common are vascular or diabetic ulcers, drepanocytosis, yaws, ecthyma, phagedenic ulcers, chronic herpes or all other infectious ulcers, as well as skin cancers [9, 10]. The differential clinical diagnosis should also be broken down into age groups. For example, vascular ulcers are rare in children. For ulcer descriptions, most differential diagnoses proposed are based on single expert opinions and are rarely based on actual data from prospective studies [11]. Skin ulcers were infrequently seen in studies in Nigeria and Ethiopia which described the principle causes of skin problems presenting at dermatological consultation and within households [12, 13]. We aimed to describe the differential diagnoses for lesions with suspect *M*. *ulcerans* infection in central Cameroon.

## Methods

In Cameroon, cases of *M*. *ulcerans* infection have been reported in 8 regions, namely Adamawa, Centre, East, Far North, North West, West, South, and South West. The Akonolinga and the Ayos health districts located along the Nyong River were the first endemic foci described in the country [14]. In 2002 Médecins Sans Frontières, in collaboration with the Ministry of Health, began treating BU in the Akonolinga Health District, where the overall BU prevalence was 0.47% in 2007 [15].

Between 2011 and 2013, a prospective cohort study was conducted in Akonolinga Health District, central Cameroon. All individuals presenting at Akonolinga District Hospital with a skin lesion suspect of new BU (defined as nodule, plaque, localized swelling and/or an ulcer in an individual residing in or having spent at least one night in a known *M*. *ulcerans* endemic area were enrolled consecutively after written consent. Patients who reported previous medical or surgical treatment for BU were excluded. Clinical data and clinicians’ judgment on likelihood of BU (using a semi-quantitative scale with four levels: BU very likely, likely, possible, unlikely) were collected, before the results of laboratory examination were available. One experienced dermatologist from Yaounde Central Hospital visited the project every two weeks and saw a subset of the patients. Photographs were taken of all suspect lesions. Photographs were independently reviewed by two dermatologists: the first from Yaounde Central Hospital and the second, experienced in tropical skin diseases from Geneva University Hospitals (Geneva, Switzerland). Skin biopsies were also independently reviewed by two histopathologists one in Yaounde and one in Geneva, respectively.

Dry swabs from ulcerative lesions and fine-needle aspirates of non-ulcerative lesions were examined after ZN staining in Akonolinga Hospital laboratory the day they were collected. Another set of samples was sent to the reference laboratory in Yaounde (Centre Pasteur Cameroon, CPC) weekly, where, after pooling of the samples for each lesion, ZN direct examination was repeated, followed by PCR targeting IS2404 and culture. The CPC is part of a BU external quality control program run by the Institute of Tropical Medicine in Antwerp, Belgium. Two 4 mm punch skin biopsies were performed on ulcerative lesions, one at the edge and one in the center of the lesion. Biopsies were stored in 4% formaldehyde before being sent weekly to Yaounde (CPC), where slides were prepared for histopathological examination. Slides were systematically stained with hematoxylin/eosin and Ziehl-Neelsen. After a first examination in Yaounde, the slides and remaining paraffin block were sent to Geneva University Hospitals, where another reading took place.

Patients were screened for diabetes by a rapid test for plasma glucose, and for syphilis and yaws by a rapid syphilis test (SD Bioline). HIV testing was systematically proposed to all patients, after counseling. Suspect cases of sickle cell disease were tested by Emmel test and confirmed by haemoglobin electrophoresis in CPC. Patients were transferred to Yaounde for radiographs for those starting specific Buruli treatment, and for those with suspect osteomyelitis. Vascular examination through Doppler ultrasound was available in Yaounde, and was performed in case of suspicion of a vascular origin to the lesion.

Cases without a definite diagnosis after initial evaluation by the two dermatologists were discussed in consensus meetings that took place by videoconference between Yaounde and Geneva with participation of clinicians, histopathologists and dermatologists. During these meetings, all clinical information, laboratory results and wound evolution were reviewed for specific cases. Clinicians were asked to estimate the probability of BU on a scale from 0 to 10 (0 being null probability of BU and 10 being total certainty). A final diagnosis of BU named “consensus diagnosis” was defined as either: at least two positive tests among ZN done in Akonolinga, ZN or PCR in CPC, positive acid-fast bacilli (AFB) on histology; being the most likely diagnosis based on both expert reviews of photographs; or BU agreed upon as the most likely diagnosis during consensus meetings. While the main objective of these meetings was to differentiate BU from non-BU cases, final diagnoses of non-BU cases were also reviewed and agreed upon.

Statistical analysis was performed using Stata/SE 12.1 (College Station, USA). Final diagnosis was described for all lesions, and according to sex, age, and HIV-status. Frequencies by category were compared by chi-square test and results given with p-value. Final diagnoses were regrouped into predefined diagnostic categories agreed upon by the dermatologists. Results presented in this paper are restricted to diagnosis of ulcerative lesions, which represented the majority of cases.

### Ethics Statement

Ethical approval was given by the National Ethics Committee of Cameroon, the Central Commission on Human Subject Research Ethics of the Geneva University Hospital, and the Ethical Review Board of Médecins Sans Frontières. The study was also approved by the Ministry of Health, in the framework of the National Yaws, Leishmaniasis, Leprosy and Buruli Control Program, as well as by the health authorities of the Akonolinga District and Hospital administration. All patients included in the study provided informed written consent; parents/guardians provided consent on behalf of their children if participants were under the age of 18.

## Results

Between October 2011 and December 2013, 447 patients were screened and 367 included in the study. 364 were included in the final analysis (3 secondary exclusions due to missing clinical data), corresponding to 422 lesions. Of the 80 cases not included, 50 had skin lesions not suspect of BU, 26 had already been previously treated for Buruli ulcer, 3 did not consent, and one was lost-to-follow-up (left the hospital) before completing screening.

**Fig 1:**
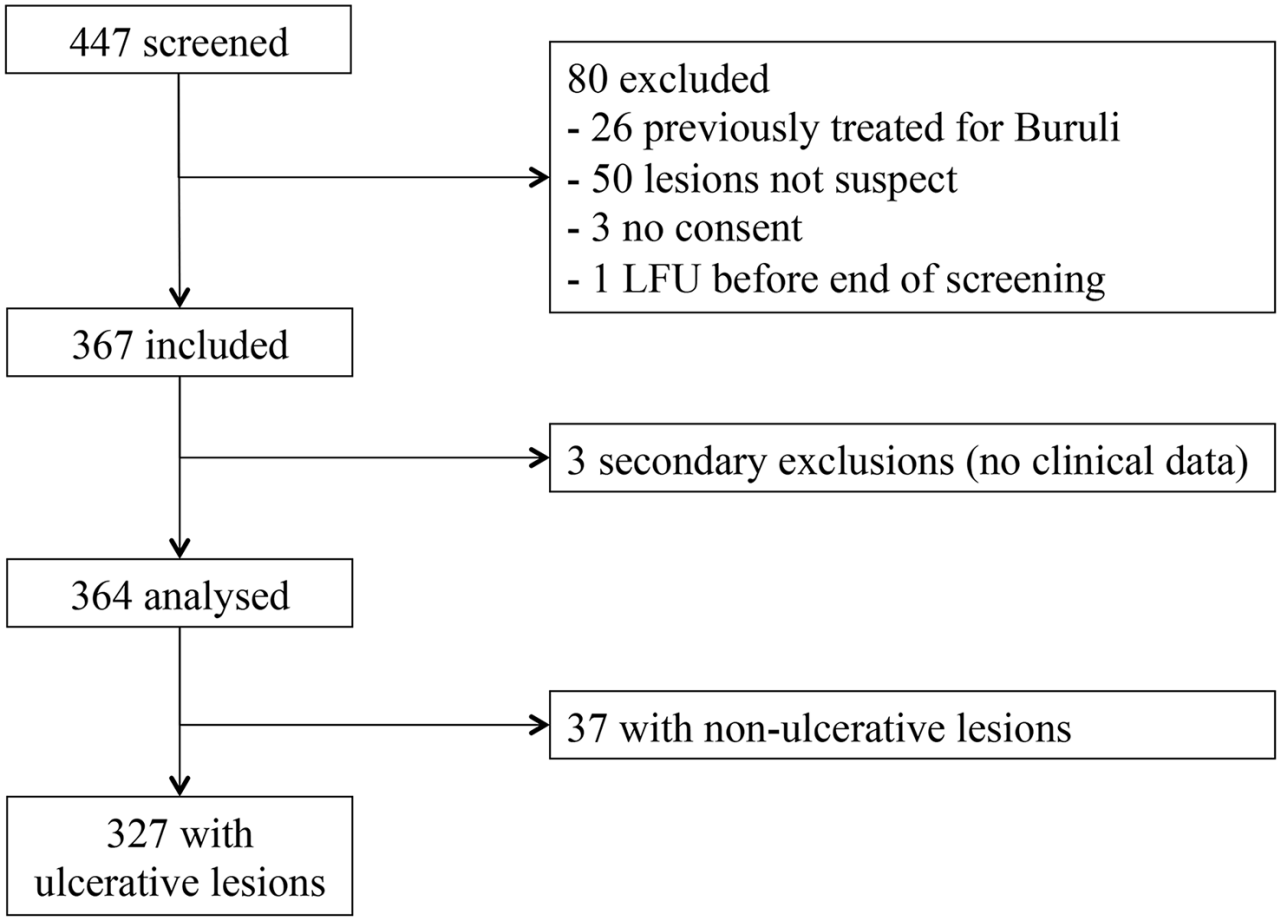
Patient flow, Buruli score study 2011–2013, Akonolinga, Cameroon.

The majority of patients (327/364 = 89.8%) presented with ulcerative lesions (Fig 1). The median age of patients with ulcers was 37 years (range 0 to 87) and 213 (65%) were males. According to WHO classification of ulcer severity, patients were of category I, II or III in 33.6%, 40.1% and 26.3% of cases, respectively.

The median symptom duration was 24 weeks (IQR 5 to 104 weeks, or 1 month to 2 years). More than half of the patients had already received traditional (57.8%), topical (65.1%) or systemic (68.5%) treatments for their lesions. A history of trauma, mainly previous injury, was mentioned by 118 (36.1%). Patients were aware of another Buruli case in their village in121 (37.0%) cases, including 42 (34.7%) who lived under the same roof as a known case. Patient characteristics and exposure history are shown in Table 1. On clinical examination, 31 (9.5%) had an axillary temperature above 37.5°C. Vascular examination was abnormal in 70 (21.3%) of the patients, the most frequently described anomalies were venous insufficiency (39/70 = 55.7%) followed by mixed insufficiency (24.3%), arterial insufficiency (10.0%), and others or not specified (10.0%).

**Table 1:** Characteristics of patients’ history. Univariate analysis in patients with ulcerative lesions. Buruli score study 2011–2013, Akonolinga, Cameroon.

	Total (N = 327)		BU (N = 87)		Non-BU (N = 240)		
	n	%	n	%	n	%	p-value*
**Median duration of present episode,** in weeks (IQR)	23	(5–104)	12	(4–24)	36	(6–144)	<0.001
Missing	7		0		7		
**Other treatments received:**							
Topical	213	65.1	49	56.3	164	68.3	0.044
Systemic	224	68.5	48	55.2	176	73.3	0.002
traditional	189	57.8	48	55.2	141	58.8	0.563
**History of trauma**	118	36.1	26	29.9	61	70.1	0.16
*Type of trauma*							0.841
*Injury*	*53*	*45*.*7*	*11*	*42*.*3*	*42*	*46*.*7*	
*Insect bite*	*11*	*9*.*5*	*3*	*11*.*5*	*8*	*8*.*9*	
*Burn*	*13*	*11*.*2*	*2*	*7*.*7*	*11*	*12*.*2*	
*Others*	*39*	*33*.*6*	*10*	*38*.*5*	*29*	*32*.*2*	
*Missing*	*2*						
**BU cases in the vicinity (1 missing)**	121	37.0	40	46.0	81	33.8	0.043
*Living in the same village*	*77*	64.7	25	62.5	52	65.8	
*Living under the same roof*	*42*	35.3	15	37.5	27	34.2	
*Missing*	*2*						
**Median age**, in years (IQR)	37	(18–56)	20	(10–34)	42	(23–57)	<0.001
**Gender**							0.005
Male	213	65.1	46	52.9	167	69.6	
Female	114	34.9	41	47.1	73	30.4	

* p-value comparing BU and non-BU (ranksum test for continuous and chi^2^ for categorical variables)

Sixty-three patients (19.3%) were HIV-positive (the 7 not tested and one with a discordant result were considered HIV-negative), with a median CD4 count of 362 (IQR 210–653; 12 missing CD4 count). Hypertension was confirmed in only 4 cases (1.2%) and suspected in another 9 (2.8%). Diabetes was suspected in 22 (6.7%) and confirmed 7 (2.1%) cases, respectively. Sickle cell disease was confirmed in 6 (1.8%) patients. Rapid syphilis test was positive in 48/327 patients (14.7%) overall, 5.9% (4/68; 2 missing) among children under 15 years and 17.4% (44/253; 4 missing) among adults. Of the patients with a positive rapid test, 39 were further tested with TPHA and VDRL. Three patients had a positive VDRL and TPHA, all were older than 15 years of age. Ten were negative for both VDRL and TPHA, including the 4 children, likely indicating a false positive rapid test. 26 were VDRL negative and TPHA positive, reflecting an old infection.

Clinician 1 saw 160 lesions, clinician 2 evaluated 230 other lesions and dermatologist in the field saw 218 lesions. The two dermatologists gave their opinion on 327 lesions. *M ulcerans* was judged very likely between 11.3% (18/160) and 31.3% (72/230) of patients with ulcerative lesions according to the clinicians, 25.2% (55/218) of the patients seen by the dermatologist, and 16.8% (55/327) and 26.9% (88/327) of the opinions given on photographs. Concordance of clinical likelihood of BU and dermatological evaluation based on photographs graded in four levels were poor (kappa = 0.25 and 0.20, respectively). Combining the four into two levels (“very likely and likely” versus “possible and unlikely”) increased concordance to 0.46 and 0.40, respectively, with agreement in 64% and 72% of the cases, respectively. Laboratory tests were positive for BU in 8.9% (29/327) of culture, 10.7% (35/327) and 17.7% (58/327) for ZN done in Yaounde and Akonolinga, respectively, and 23.6% (77/327) for PCR. Apart from specific cases of neoplasia, histopathology was of little help when determining the final diagnosis, with 37% (131/350) and 54% (187/350) lesions receiving histopathological diagnoses of non-specific ulcers in Yaounde and Geneva, respectively.

Out of 327 patients, two thirds (219) could be classified as BU or non-BU based on the laboratory test results and evaluations made on photographs. There was agreement on the final diagnoses of the non-BU cases between the two independent dermatologists. The remaining 108 patients with 125 controversial lesions were discussed during eight consensus meetings, attended by 3 to 6 clinicians, 2 dermatologists and 2 histopathologists. The mean BU probability for lesions considered to be BU by the clinicians was 8.4 (SD 0.7), and 2.8 (SD 1.4) for those considered non-BU. Discrepant non-BU diagnoses were also reviewed during these meetings.

*M ulcerans* was considered the final diagnosis by consensus in 26.6% (87/327) patients. Of those, 74 (85.1%) were confirmed cases with at least one positive laboratory test (ZN, PCR or culture). The second and third most frequently detected ulcers were venous ulcers (12.8%) followed by superficial bacterial skin infections (ecthyma, impetigo, folliculitis, or boils) (11.9%). Other frequent diagnoses (>5% of patients) were mixed vascular ulcers (6.7%), post-traumatic (7.3%), erysipelas (6.4%), and osteomyelitis (5.8%). Sixteen patients (4.9%) had non-specific ulcers that could not be attributed to a clear etiology. Seventeen cases of neoplasia were found. Detailed diagnoses are shown in Table 2.

**Table 2:** Differential diagnosis of patients with ulcerative skin lesions, Buruli score study 2011–2013, Akonolinga, Cameroon.

Diagnostic category	(N = 327)	
Detailed diagnosis	n	%
*M ulcerans* infection	87	26.6
Vascular and neuropathic ulcers		
Subtotal	73	22.3
Venous ulcer	42	12.8
Mixed vascular ulcer*	22	6.7
Neuropathic ulcer	7	2.1
Arterial ulcer	1	0.3
Pressure sore	1	0.3
Bacterial infections		
Subtotal	68	20.8
Ecthyma /impetigo/boils	39	11.9
Infectious dermohypodermatitis	21	6.4
Abcess	3	0.9
Necrotizing fasciitis	2	0.6
Phagedenic ulcer	2	0.6
Gum	1	0.3
Post-traumatic ulcers		
Subtotal	25	7.6
Post-traumatic	24	7.3
Toxic necrosis	1	0.3
Osteomyelitis		
Subtotal	19	5.8
Osteomyelitis	19	5.8
Neoplasia		
Subtotal	17	5.2
Carcinoma*	7	2.1
Kaposi	7	2.1
Sarcoma	3	0.9
Inflammatory diagnoses		
Subtotal	9	2.8
Vasculitis	5	1.5
Neutrophilic dermatosis (pyoderma gangrenosum)	2	0.6
Eczema	2	0.6
Hemopathies and other systemic diseases		
Subtotal	8	2.4
Ulcer of hemopathies	7	2.1
Chronic oedema due to cardiac insufficiency	1	0.3
Benign disorders		
Subtotal	2	0.6
Insect bite	1	0.3
Benign tumor	1	0.3
Non-bacterial infections		
Subtotal	2	0.6
Oedema associated with parasite (filaria)	1	0.3
Herpes	1	0.3
Fistulas		
Subtotal	2	0.6
Abdominal fistula	1	0.3
Anal fistula	1	0.3
Non-specific ulcers		
Subtotal	16	4.9
Non-specific ulcer	16	4.9

* One patient had two lesions with two different final diagnoses (venous ulcer and squamous cell carcinoma), counted in both categories

The proportion of BU was the same between HIV-positive and HIV-negative patients (27.0% versus 26.5%; p = 0.940). HIV-positive patients were more likely to present with large lesions (29.1% above 15 cm vs. 14.6% p = 0.01). Bacterial infections were more frequent among HIV positive patients (34.9% vs. 17.4%; p = 0.002) while vascular or neuropathic ulcers (mainly venous or mixed vascular ulcers) were more frequent among HIV-negative patients (25.0% vs. 17.4%; p = 0.017). Half of children under 15 years of age had BU, compared to 26.8% and 13.9% among patients 15 to 44 years of age and patients ≥45 years of age, respectively (p<0.001). Among children, other frequent diagnoses were superficial bacterial skin infections (24.3%) and osteomyelitis (11.4%). When compared to BU, both vascular ulcers and bacterial infections were more frequently reported among patients ≥45 years old (40.0% and 19.2% vs. 13.9%; p< 0.001 and p = 0.081, respectively). Half of the vascular or neuropathic ulcers (36/73) were confirmed by Doppler examination. The absolute numbers of *M ulcerans* infections was similar between males and females (46 vs. 41). However, because there were more males than females included in the study (213 vs 114), when included, females had a higher chance of having a lesion diagnosed as BU (36.0% vs. 21.6% for males; p = 0.005). Compared to females, males more frequently presented with other causes of skin lesions, particularly all vascular lesions (venous ulcers, hemopathies, vasculitis), as well as post-traumatic and osteomyelitis (Table 3).

**Table 3:** Category of final diagnosis of patients with ulcerative skin lesions, by HIV status, age and gender, Buruli score study 2011–2013, Akonolinga, Cameroon.

	Total		By age						By gender				By HIV status			
			Below 15		15 to 44		45 and above		Male		Female		Positive		Negative or not tested	
	(N = 327)		(N = 70)		(N = 127)		(N = 130)		(N = 213)		(N = 114)		(N = 63)		(N = 264)	
*M ulcerans* infection	87	26.6	35	50.0	34	26.8	18	13.9	46	21.6	41	36.0	17	27	70	26.5
Vascular and neuropathic ulcers*	73	22.3	1	1.4	20	15.8	52	40.0	56	26.3	17	14.9	7	11.1	66	25
Bacterial infections	68	20.8	17	24.3	26	20.5	25	19.2	38	17.8	30	26.3	22	34.9	46	17.4
Post-traumatic ulcers	25	7.7	4	5.7	9	7.1	12	9.2	20	9.4	5	4.4	3	4.8	22	8.3
Osteomyelitis	19	5.8	8	11.4	6	4.7	5	3.9	15	7.0	4	3.5	3	4.8	16	6.1
Neoplasia*	17	5.2	1	1.4	7	5.5	9	6.9	10	4.7	7	6.1	4	6.4	13	4.9
Inflammatory diagnoses	9	2.8	0	0.0	6	4.7	3	2.3	9	4.2	0	0.0	1	1.6	8	3
Hemopathies and other systemic diseases	8	2.5	1	1.4	6	4.7	1	0.8	7	3.3	1	0.9	0	0	8	3
Benign disorders	2	0.6	1	1.4	1	0.8	0	0.0	1	0.5	1	0.9	0	0	2	0.8
Non-bacterial infections	2	0.6	0	0.0	2	1.6	0	0.0	2	0.9	0	0.0	1	1.6	1	0.4
Fistulas	2	0.6	1	1.4	1	0.8	0	0.0	2	0.9	0	0.0	0	0	2	0.8
Non-specific ulcers	16	4.9	1	1.4	9	7.1	6	4.6	8	3.8	8	7.0	5	7.9	11	4.2

* One patient had two lesions with two different final diagnoses (venous ulcer and squamous cell carcinoma), counted in both categories

## Discussion

We describe the differential diagnosis of Buruli ulcer in an area endemic for *M*. *ulcerans*, based on prospectively collected data. The strength of our results is based on a rigorous consensus approach that combines clinical diagnosis, laboratory tests and multiple expert opinion.

BU was not surprisingly the most frequent diagnosis for patients with ulcerative lesions that were included in the study. The second most frequent diagnosis category was vascular lesions (including neuropathic ulcers), followed by bacterial infections. Classical “tropical” diagnoses such as drepanocytosis, phagedenic ulcer, yaws or leishmaniasis were quite rare. We did not find any typical cutaneous yaws, although several treponemal tests were concomitantly positive but only among adults. Phagedenic ulcers are known to be associated with mixed bacterial infection and malnutrition [16, 17]. We did not observe malnutrition which could have led to a larger number of tropical ulcers [18]. We were surprised by the high number of venous insufficiencies, while arterial insufficiency, and risk factors such as hypertension and diabetes were rather uncommon unlike other settings in Africa [19, 20]. While common skin diseases seen in northern countries have been described in Sub-Saharan Africa, vascular ulcers were curiously not reported in the same studies [12,13]. Infectious complications of chronic ulcers such as cellulitis or osteomyelitis may mask the underlying diagnosis, or patients with leg ulcers might not seek specialized dermatological care and may be more likely to be followed in surgical consultations, or do not seek care at all. Médecins Sans Frontières BU program started in 2005 in Akonolinga, and provided tools for a global BU management including systemic antimicrobial therapy, physiotherapy and local wound care. Thus patients have been encouraged to seek treatment for wounds of different etiologies. This may have led to our great variety of diagnoses. In our cohort, age was the primary factor associated with vascular ulcers (Table 3), with a large number of relatively young adult males suffering from venous insufficiency. We investigated some of these ulcers by Doppler examination and morphological echography and often found old venous thrombosis. Predisposing genetic factors or systemic auto-immune diseases may be suspected.

Bacterial infections were common, especially among children and HIV-positive patients. Non-BU related osteomyelitis were also common, and were confirmed by X-ray evaluation. Most of them were clinically typical and were managed adequately. They were different from BU associated osteomyelitis, which was usually associated with deep BU and confirmed by positive laboratory test for BU.

Interestingly, HIV was not found to be associated with BU diagnosis among patients with skin lesions The overall HIV-prevalence (19%) appears higher than the prevalence in the general population (6.1% in Centre region in 2011)[18]. Our data do not support the hypothesis that HIV positivity is a risk factor for development of BU. However, as previously described, HIV-positive patients did present with larger lesions when compared with HIV-negative patients [21]. Patients with BU who were diagnosed with HIV received antiretroviral therapy as soon as they were diagnosed. Tumors were confirmed in 5% of all lesions. Our results appear to accurately reflect the cancer profile in Sub-Saharan Africa. Kaposi sarcoma was the most frequently diagnosed cancer with 7 cases, including three HIV- positive patients. Although AIDS associated Kaposi sarcoma is much better described, this data highlights the importance of endemic African Kaposi sarcoma [22, 23]. We found five primary epidermoid (keratinizing or not) carcinoma. In our cohort they did not complicate a Buruli ulcer. Any chronic wound is a risk factor for such a carcinoma. This is well known for vascular wounds and was exceptionally reported on Buruli ulcer [24, 25]. Histopathological analysis was crucial for confirmation of such a diagnosis.

Our study has some limitations: we lacked agreement between laboratory test and clinical diagnosis for several cases and we cannot exclude some diagnostic misclassification due to our consensus approach. In particular, BU may have been over-diagnosed by the experts. However, it was the most relevant approach to reach a final precise diagnosis for most cases, as shown by the limited number of non-specific ulcers. Expert dermatological opinion by dermatologists based on photographs, depends on the quality of photographs and is limited when compared to clinical diagnosis. Traditional medicine products or any topical drug applied on a wound just before sampling may have led to false negative results. Furthermore, secondary infection may delay BU diagnosis by modifying clinical presentation. Follow- up is an important step in clinical evaluation, but was not possible for several patients. The contribution of histopathology to BU diagnosis was disappointing. We would suggest keeping a small punch biopsy for differential diagnosis of atypical or complex cases, only in collaboration with an experienced laboratory. Finally, we offered treatment to patients with confirmed BU and non-BU. When non-BU management was severe and complex, for rare tumors or vasculitis, for example, patients who found it difficult to understand the importance of additional investigations and treatment were frequently lost to follow-up. Interestingly, we found that some other parameters that were thought to be typical of Buruli were instead typical of vascular ulcers or bacterial infection. Management of chronic wounds is usually challenging and requires a multidisciplinary approach. Even in an endemic area the large majority of wounds are not associated with BU and we advocate for a syndromic approach based on improved diagnosis and adapted treatment for chronic wounds. A program and specific education for health care professionals on chronic wound diagnosis and care has been reinforced in Cameroon [26].

### Conclusion

We highlight the differential diagnosis of skin lesions in a Buruli endemic area of Cameroon. Most skin lesions were not actually due to *M ulcerans*. Vascular and bacterial causes were very common, while typically “tropical” diagnoses were rare. When making such diagnoses tumors and osteomyelitis should not be overlooked. Age, sex and HIV status need to be taken into account when assessing patients with skin lesions.

## Supporting Information

S1 Checklist STROBE checklist. (DOC)
