# Supplementary material for: Differential Diagnosis of Skin Ulcers in a Mycobacterium ulcerans Endemic Area: Data from a Prospective Study in Cameroon
Source: PLoS Negl Trop Dis. 2016 Apr 13;10(4):e0004385. doi: 10.1371/journal.pntd.0004385 (PMC4830608; doi:10.1371/journal.pntd.0004385)
Supplement: S1 Checklist — (DOC) [file pntd.0004385.s001.doc]

STROBE Statement—Checklist of items that should be included in reports of ***cohort studies***

|  | Item No | Recommendation |
| --- | --- | --- |
| **Title and abstract** | 1 | a)Indicate the study’ design with a commonly used term in the title or the abstract  *Prospective study describing differential diagnosis of Mycobacterium ulcerans infection in a cohort of individuals with ulcerative lesions, Cameroon.* |
|  |  | (*b*) Provide in the abstract an informative and balanced summary of what was done and what was found  *Done in abstract* |
| Introduction | | |
| Background/rationale | 2 | Explain the scientific background and rationale for the investigation being reported  *Cases of painless ulcerated plaques with undermined edges, classically described as BU, are believed to be relatively easy to clinically diagnose in endemic regions. Akonolinga in Cameroon is known as a BU endemic area. Some forms of BU can be mistaken for other types of ulcers found in tropical areas. Laboratory testing confirms diagnosis but may be expensive and laboratories far from care centers*. *In Africa very few cohort studies describe the various diagnosis of skin lesions and particularly skin ulcers.* |
| Objectives | 3 | State specific objectives, including any prespecified hypotheses  *Therefore, we aimed to describe the differential diagnoses for ulcerative lesions with suspect M. ulcerans infection in central Cameroon* |
| Methods | | |
| Study design | 4 | Present key elements of study design early in the paper  *Between 2011 and 2013, a prospective cohort study was conducted in Akonolinga Health District, central Cameroon* |
| Setting | 5 | Describe the setting, locations, and relevant dates, including periods of recruitment, exposure, follow-up, and data collection  *The Akonolinga and the Ayos health districts located along the Nyong River were the first endemic foci described in the country [14]. In 2002 Médecins Sans Frontières, in collaboration with the Ministry of Health, began treating BU in the Akonolinga Health District, where the overall BU prevalence was 0.47% in 2007 . Study was conducted between 2011 and 2013.* *Patients were cared for in accordance with MSF protocol in effect in Akonolinga. Pending the results of investigations, all patients suspected of infection with MU received appropriate wound care and additional investigations (xray, Doppler echography) and treatment if required. If investigations revealed another diagnosis than MU, the patient was referred to the Akonolinga District Hospital* |
| Participants | 6 | a)Give the eligibility criteria, and the sources and methods of selection of participants. Describe methods of follow-up  *All individuals presenting at Akonolinga District Hospital with a skin lesion suspect of new BU (defined as nodule, plaque, localized swelling and/or an ulcer in an individual residing in or having spent at least one night in a known M. ulcerans endemic area ) were enrolled consecutively.* ***See page 6*** |
| Variables | 7 | Clearly define all outcomes, exposures, predictors, potential confounders, and effect modifiers. Give diagnostic criteria, if applicable  ***See p7 and table 1and 2****.*  *Clinical evaluation by clinician and dermatologists, photographs evaluation and laboratory tests results (zhiel Nielsen(ZN), culture, PCR, histology) and for complex cases diagnosis conclusion was established in consensus meeting including clinicians from the field, expert dermatologists and histopathologists. While the main objective of these meetings was to differentiate BU from non-BU cases, final diagnoses of non-BU cases were also reviewed and agreed upon.*  ***Diagnostic criteria****: A final diagnosis of MU infection was defined as at least two positive among ZN done Akonolinga, ZN or PCR in CPC, positive BAAR on histology OR MU being the most likely diagnosis based on both expert reviews of photographs OR MU agreed upon as the most likely diagnosis during consensus meetings* |
| Data sources/ measurement | 8* | For each variable of interest, give sources of data and details of methods of assessment (measurement). Describe comparability of assessment methods if there is more than one group  *Data were collected on a specific case report form. Data from the medical history and clinical examination were collected from the medical record used at initial examination. Clinical lesions were measured using a centimeter scale in the direction of the longest diameter through the center of the lesion. The results of laboratory tests were directly recorded in the case report form.*  *Data were entered from the paper questionnaire into an Epidata database developed specifically for the project.*  See variables P7 and table 1 |
| Bias | 9 | Describe any efforts to address potential sources of bias  *This study was prospective. Médecins sans Frontières and Ministry of Health have been used to organized information campaigns for detection and care of M ulcerans infection. Patients came by their own when they had a wound or persistent skin lesion*. *There were 2 different clinicians working at different period for the first evaluation which may have been a bias. Few delay were observed between tests results and clinical diagnosis. The 2 dermatologists saw all photographs and consensus meeting helped to clinical difficulties.* |
| Study size | 10 | Explain how the study size was arrived at  *Based on previous studies and potential recruitment in this district hospital we aimed at all 500 cases over 2 years. Around 120 confirmed BU cases were expected.* |
| Quantitative variables | 11 | Explain how quantitative variables were handled in the analyses. If applicable, describe which groupings were chosen and why |
| Statistical methods | 12 | a)Describe all statistical methods, including those used to control for confounding  (*b*) Describe any methods used to examine subgroups and interactions  *Statistical analysis was performed using Stata/SE 12.1 (College Station, USA). Final diagnosis was described for all lesions, and according to sex, age, and HIV-status. Frequencies by category were compared by chi-square test and results given with p-value. Final diagnoses were regrouped into predefined diagnostic categories agreed upon by the dermatologists. Results presented in this paper are restricted to diagnosis of ulcerative lesions, which represented the majority of cases. See* ***p7*** |
|  |  | (*c*) Explain how missing data were addressed  *Patients with missing data were excluded. +* ***Fig.1*** |
|  |  | (*d*) If applicable, explain how loss to follow-up was addressed  ***Figure 1*** |
|  |  | (*e*) Describe any sensitivity analyses.  *Descriptive study.* |
| Results | | |
| Participants | 13* | a)Report numbers of individuals at each stage of study—eg numbers potentially eligible, examined for eligibility, confirmed eligible, included in the study, completing follow-up, and analysed  (b) Give reasons for non-participation at each stage  *447 patients were screened and 367 included in the study. 364 were included in the final analysis (3 secondary exclusions due to missing clinical data), corresponding to 422 lesions. Of the 80 cases not included, 50 had skin lesions not suspect of BU, 26 had already been treated previously treated for Buruli ulcer, 3 did not consent, and one was lost-to-follow-up (left the hospital) before completing screening.*  *The majority of patients (327/364=89.8%) presented with ulcerative lesions*  *M ulcerans* *was judged very likely between 11.3% (18/160) and 31.3% (72/230) of patients with ulcerative lesions according to the clinicians, 25.2% (55/218) of the patients seen by the dermatologist, and 16.8% (55/327) and 26.9% (88/327) of the opinions given on photographs.* ***Pages 11-12*** |
|  |  | (c) Consider use of a flow diagram.  *Figure 1* |
| Descriptive data | 14* | a)Give characteristics of study participants (eg demographic, clinical, social) and information on exposures and potential confounders  Table 1 and see p6-7  *Sixty-three patients (19.3%) were HIV-positive (the 7 not tested and one with a discordant result were considered HIV-negative), with a median CD4 count of 362 (IQR 210 – 653; 12 missing CD4 count). Hypertension was confirmed in only 4 cases (1.2%) and suspected in another 9 (2.8%). Diabetes was suspected in 22 (6.7%) and confirmed 7 (2.1%) cases, respectively. Sickle cell disease was confirmed in 6 (1.8%) patients. Rapid syphilis test was positive in 48/327 patients (14.7%) overall, 5.9% (4/68; 2 missing) among children under 15 years and 17.4% (44/253; 4 missing) among adults. Of the patients with a positive rapid test, 39 were further tested with TPHA and VDRL. Three patients had a positive VDRL and TPHA, all were older than 15 years of age. Ten were negative for both VDRL and TPHA, including the 4 children, likely indicating a false positive rapid test. 26 were VDRL negative and TPHA positive, reflecting an old infection.* |
|  |  | (b) Indicate number of participants with missing data for each variable of interest |
|  |  | (c) Summarise follow-up time (eg, average and total amount):  *Not the aim. Only diagnosis* |
| Outcome data | 15* | Report numbers of outcome events or summary measures over time |
| Main results | 16 | a)Give unadjusted estimates and, if applicable, confounder-adjusted estimates and their precision (eg, 95% confidence interval). Make clear which confounders were adjusted for and why they were included  *M ulcerans was considered the final diagnosis by consensus in 26.6% (87/327) patients. Of those, 74 (85.1%) were confirmed cases with at least one positive laboratory test (ZN, PCR or culture). The second and third most frequently detected ulcers were venous ulcers (12.8%) followed by ecthyma or pyodermatitis (11.9%). Main differential diagnosis detailed in Table 2 . p11-12* |
|  |  | (*b*) Report category boundaries when continuous variables were categorized |
|  |  | (*c*) If relevant, consider translating estimates of relative risk into absolute risk for a meaningful time period |
| Other analyses | 17 | Report other analyses done—eg analyses of subgroups and interactions, and sensitivity analyses.  *Laboratory test sensitivity*: *not detailed in this paper. We present here the lab tests as an important criteria for final diagnosis.* *M ulcerans was considered the final diagnosis by consensus in 26.6% (87/327) patients. Of those, 74 (85.1%) were confirmed cases with at least one positive laboratory test (ZN, PCR or culture)*  ***Table 3****. Sub group diagnosis and patients comparison by Age, sex and HIV status.****p15***  *HIV: The proportion of BU was the same between HIV-positive and HIV-negative patients (27.0% versus 26.5%; p= 0.940)…..*  *Age: Half of children under 15 years of age had BU, compared to 26.8% and 13.9% among patients 15 to 44 years of age and patients ≥45 years years of age, respectively (p<0.001)….*  *Sex: The absolute numbers of M ulcerans infections was similar between males and females (46 vs. 41)…..* |
| Discussion | | |
| Key results | 18 | Summarise key results with reference to study objectives  *BU was the most frequent diagnosis for patients with ulcerative lesions. The second most frequent diagnosis category was vascular lesions (including neuropathic ulcers), followed by bacterial infections. Classical “tropical” diagnoses such as drepanocytosis, phagedenic ulcer, yaws or leishmaniasis were quite rare.* ***See table 2 and 3***  *The proportion of non-BU ulcerative lesion was important and differential diagnosis various. The frequency of vascular lesions was particularly of interest.* |
| Limitations | 19 | Discuss limitations of the study, taking into account sources of potential bias or imprecision. Discuss both direction and magnitude of any potential bias  *We lacked agreement between laboratory test and clinical diagnosis for several cases and we cannot exclude some diagnostic misclassification due to our consensus approach. In particular, BU may have been over-diagnosed by the experts.* ***P19*** |
| Interpretation | 20 | Give a cautious overall interpretation of results considering objectives, limitations, multiplicity of analyses, results from similar studies, and other relevant evidence  *We confirmed a higher prevalence of BU among children than in adults as it is already described in Western Africa. In children bacterial infections including osteomyelitis is the second most frequent diagnosis. The analyse by age shows that older adults present lesion of vascular origin and more skin cancers are also observed. More Males suffer from ulcer of venous origin. We suggest that vascular ulcer in adults and elderly is a frequent differential diagnosis in Africa and is neglected at different levels for different reasons such as its chronicity and management constraints: investigations, surgery, dressing, contention etc.*  *We confirm a high prevalence of HIV infection compare to the official prevalence reported by the Cameroon government. We could show that HIV patients had more severe BU lesions as it was reported in reference 21* . **p 20** |
| Generalisability | 21 | Discuss the generalisability (external validity) of the study results  Our results show a large variety of skin ulcers in a tropical area. A diagnosis of BU has to be suspected in a known endemic area. Age, sex and HIV status are factors which may help the clinician to diagnose chronic ulcers. Epidemiological studies and/or at least, management recommendations should be developed for vascular ulcers in Africa. |
| Other information | | |
| Funding | 22 | Give the source of funding and the role of the funders for the present study and, if applicable, for the original study on which the present article is based  *This study was supported by Médecins sans Frontiers, Operational Center.*  *The University Hospital of Geneva provided support for local ethical committee costs, histopathology supplementary analyses (cancers diagnosis) and dermatologist travel to Cameroon (study preparation period)* |

*Give information separately for exposed and unexposed groups.

**Note:** An Explanation and Elaboration article discusses each checklist item and gives methodological background and published examples of transparent reporting. The STROBE checklist is best used in conjunction with this article (freely available on the Web sites of PLoS Medicine at http://www.plosmedicine.org/, Annals of Internal Medicine at http://www.annals.org/, and Epidemiology at http://www.epidem.com/). Information on the STROBE Initiative is available at http://www.strobe-statement.org.
